# Supplementary material for: Antibiotic resistance is lower in Staphylococcus aureus isolated from antibiotic-free raw meat as compared to conventional raw meat
Source: PLoS One. 2018 Dec 10;13(12):e0206712. doi: 10.1371/journal.pone.0206712 (PMC6287829; doi:10.1371/journal.pone.0206712)
Supplement: S1 Table — Disk diffusion results are listed for all antibiotics except for oxacillin and vancomycin, for which standard inhibitory concentrations are listed. Dark Grey = resistant, light grey = intermediate, white = susceptible, black = MDR isolate. Those with complete resistance to at least three antibiotics were considered “multiple drug-resistant” (MDR). AFC = antibiotic-free chicken, AFT = antibiotic-free turkey, Cld = clindamycin, Tet = tetracycline, Cef = cefotaxime, Ery = erythromycin, Chl = chloramphenicol, Gen = gentamicin, Cip = ciprofloxacin, Rif = rifampin, Oxa = oxacillin, Van = vancomycin, SE = standard error, Susc = susceptible, Inter = intermediate resistance, Resis = complete resistance. All values for disk diffusion are in millimeters, which reflect the diameter of the growth ring around the antibiotic disk. (DOCX) [file pone.0206712.s001.docx]

**Supplemental Table 1: Resistance of conventional meat SA isolates for ten common antibiotics**

| **Strain** | Cld | Tet 30μg | Cef 30μg | Ery | Chl | Gen | Cip | Rif | Oxa | Van | MDR |
| --- | --- | --- | --- | --- | --- | --- | --- | --- | --- | --- | --- |
|  | 2μg |  |  | 15μg | 30μg | 10μg | 5μg | 5μg | 4μg/mL |  |  |
| B8 | 28 | 10 | 14 | 10 | 16 | 24 | 22 | 30 | Resis | Susc |  |
|  |  |  |  |  |  |  |  |  |  |  |  |
| C1 | 12 | 14 | 12 | 30 | 10 | 16 | 28 | 30 | Resis | Susc |  |
| C2 | 20 | 14 | 14 | 14 | 16 | 20 | 10 | 22 | Resis | Susc |  |
| C3 | 10 | 18 | 11 | 10 | 14 | 18 | 26 | 22 | Resis | Susc |  |
| C4 | 46 | 10 | 38 | 24 | 20 | 22 | 26 | 30 | Susc | Susc |  |
| C6 | 22 | 22 | 10 | 10 | 16 | 20 | 24 | 22 | Susc | Susc |  |
| C7 | 24 | 10 | 28 | 25 | 20 | 26 | 22 | 32 | Resis | Susc |  |
| C9 | 10 | 22 | 17 | 16 | 24 | 22 | 28 | 28 | Susc | Susc |  |
| C10 | 20 | 10 | 10 | 10 | 16 | 18 | 22 | 30 | Susc | Susc |  |
| C11 | 14 | 22 | 14 | 14 | 18 | 28 | 24 | 26 | Resis | Susc |  |
| C14 | 12 | 22 | 20 | 10 | 22 | 16 | 20 | 28 | Resis | Susc |  |
| C15 | 14 | 16 | 18 | 18 | 20 | 16 | 24 | 32 | Resis | Susc |  |
| C20 | 22 | 20 | 10 | 10 | 14 | 16 | 22 | 32 | Resis | Susc |  |
| **Chicken Mean** | 18.83 | 16.67 | 16.83 | 15.92 | 17.5 | 19.83 | 23 | 27.83 |  |  |  |
| **Chicken SE** | 2.87 | 1.44 | 2.46 | 2.01 | 1.13 | 1.17 | 1.38 | 1.14 |  |  |  |
| P3 | 12 | 20 | 26 | 16 | 30 | 26 | 32 | 22 | Resis | Susc |  |
| P4 | 10 | 24 | 16 | 14 | 22 | 18 | 10 | 24 | Resis | Susc |  |
| P5 | 40 | 28 | 42 | 14 | 22 | 30 | 30 | 32 | Resis | Susc |  |
| P6 | 30 | 10 | 18 | 10 | 28 | 24 | 32 | 38 | Resis | Susc |  |
| P7 | 10 | 10 | 10 | 10 | 16 | 20 | 22 | 20 | Susc | Susc |  |
| P9 | 54 | 32 | 22 | 10 | 26 | 30 | 28 | 32 | Susc | Susc |  |
| P10 | 20 | 10 | 20 | 26 | 20 | 18 | 26 | 28 | Susc | Susc |  |
| P11 | 10 | 10 | 26 | 16 | 16 | 16 | 24 | 24 | Resis | Susc |  |
| P12 | 12 | 20 | 24 | 18 | 10 | 20 | 24 | 22 | Susc | Susc |  |
| P13 | 20 | 10 | 18 | 18 | 18 | 24 | 16 | 26 | Susc | Susc |  |
| P14 | 20 | 12 | 18 | 20 | 22 | 10 | 16 | 32 | Resis | Susc |  |
| **Pork Mean** | 21.64 | 16.91 | 21.82 | 15.64 | 20.91 | 21.45 | 23.64 | 27.27 |  |  |  |
| **Pork SE** | 4.32 | 2.49 | 2.46 | 1.47 | 1.76 | 1.83 | 2.15 | 1.69 |  |  |  |
| T2 | 10 | 10 | 11 | 10 | 18 | 10 | 28 | 26 | Resis | Susc |  |
| T4 | 16 | 10 | 18 | 16 | 20 | 22 | 22 | 28 | Resis | Inter |  |
| T5 | 22 | 24 | 18 | 18 | 20 | 24 | 26 | 26 | Resis | Susc |  |
| T6 | 36 | 34 | 14 | 18 | 18 | 24 | 22 | 28 | Resis | Susc |  |
| T7 | 40 | 16 | 24 | 10 | 18 | 22 | 24 | 24 | Resis | Susc |  |
| T10 | 30 | 28 | 24 | 22 | 14 | 20 | 26 | 30 | Resis | Susc |  |
| T12 | 14 | 22 | 12 | 10 | 14 | 16 | 22 | 20 | Susc | Inter |  |
| T13 | 18 | 24 | 10 | 12 | 18 | 16 | 10 | 22 | Susc | Susc |  |
| T14 | 24 | 20 | 22 | 10 | 16 | 16 | 10 | 26 | Resis | Susc |  |
| **Turkey Mean** | 23.33 | 20.89 | 17.0 | 14.0 | 17.33 | 18.89 | 21.11 | 25.56 |  |  |  |
| **Turkey SE** | 3.40 | 2.65 | 1.84 | 1.53 | 0.75 | 1.57 | 2.21 | 1.04 |  |  |  |
|  |  |  |  |  |  |  |  |  |  |  |  |
| **Total Mean** | 21.27 | 17.70 | 18.45 | 15.12 | 18.55 | 20.24 | 22.67 | 27.09 |  |  |  |
| **Total SE** | 1.98 | 1.24 | 1.34 | 0.97 | 0.78 | 0.86 | 1.04 | 0.75 |  |  |  |

Disk diffusion results are listed for all antibiotics except for oxacillin and vancomycin, for which standard inhibitory concentrations are listed. Dark Grey = resistant, light grey = intermediate, white = susceptible, black = MDR isolate. Isolates with complete resistance to at least three antibiotics were considered “multiple drug-resistant” (MDR). B=beef, C=chicken, P=pork, T=turkey, Cld=clindamycin, Tet=tetracycline, Cef=cefotaxime, Ery=erythromycin, Chl=chloramphenicol, Gen=gentamicin, Cip=ciprofloxacin, Rif=rifampin, Oxa=oxacillin, Van=vancomycin, SE=standard error, Susc=susceptible, Inter=intermediate resistance, Resis=complete resistance. All values for disk diffusion are in millimeters, which reflect the diameter of the growth ring around the antibiotic disk. Two chicken SA isolates and 1 turkey isolate were only tested for prevalence/genotyping and not by disk diffusion, which is why sample numbers differ for this table compared to other parts of the manuscript.
