# Supplementary material for: Antibiotic resistance is lower in Staphylococcus aureus isolated from antibiotic-free raw meat as compared to conventional raw meat
Source: PLoS One. 2018 Dec 10;13(12):e0206712. doi: 10.1371/journal.pone.0206712 (PMC6287829; doi:10.1371/journal.pone.0206712)
Supplement: S2 Table — Disk diffusion results are listed for all antibiotics except for oxacillin and vancomycin, for which standard inhibitory concentrations are listed. Dark Grey = resistant, light grey = intermediate, white = susceptible, black = MDR isolate. Isolates with complete resistance to at least three antibiotics were considered “multiple drug-resistant” (MDR). B = beef, C = chicken, P = pork, T = turkey, Cld = clindamycin, Tet = tetracycline, Cef = cefotaxime, Ery = erythromycin, Chl = chloramphenicol, Gen = gentamicin, Cip = ciprofloxacin, Rif = rifampin, Oxa = oxacillin, Van = vancomycin, SE = standard error, Susc = susceptible, Inter = intermediate resistance, Resis = complete resistance. All values for disk diffusion are in millimeters, which reflect the diameter of the growth ring around the antibiotic disk. Two chicken SA isolates and 1 turkey isolate were only tested for prevalence/genotyping and not by disk diffusion, which is why sample numbers differ for this table compared to other parts of the manuscript. (DOCX) [file pone.0206712.s002.docx]

**Supplemental Table 2: Resistance of antibiotic-free meat SA isolates for ten common antibiotics**

| **Strain** | Cld | Tet 30μg | Cef 30μg | Ery | Chl | Gen | Cip | Rif | Oxa | Van | MDR |
| --- | --- | --- | --- | --- | --- | --- | --- | --- | --- | --- | --- |
|  | 2μg |  |  | 15μg | 30μg | 10μg | 5μg | 5μg | 4μg/mL | 6μg/mL |  |
| AFC10 | 26 | 14 | 20 | 20 | 19 | 14 | 20 | 24 | Susc | Susc |  |
| AFC12 | 30 | 12 | 26 | 30 | 19 | 19 | 20 | 24 | Susc | Susc |  |
| AFC17 | 31 | 10 | 24 | 34 | 26 | 28 | 24 | 26 | Susc | Susc |  |
| AFC19 | 28 | 10 | 22 | 34 | 21 | 24 | 26 | 26 | Susc | Susc |  |
| AFC20 | 24 | 12 | 24 | 28 | 21 | 20 | 24 | 28 | Susc | Susc |  |
| AFC23 | 24 | 20 | 24 | 28 | 19 | 16 | 24 | 30 | Susc | Susc |  |
| AFC24 | 30 | 16 | 24 | 30 | 22 | 22 | 18 | 30 | Susc | Susc |  |
| **AF Chicken Mean** | 27.6 | 13.4 | 24 | 29.1 | 21 | 20.4 | 22.3 | 26.9 |  |  |  |
| **AF Chicken SE** | 1.1 | 1.4 | 0.72 | 1.8 | 0.95 | 1.8 | 1.1 | 0.96 |  |  |  |
| AFT2 | 28 | 20 | 30 | 10 | 26 | 31 | 26 | 28 | Susc | Susc |  |
| AFT10 | 30 | 30 | 28 | 20 | 24 | 22 | 28 | 20 | Susc | Susc |  |
| AFT15 | 24 | 14 | 24 | 26 | 20 | 19 | 26 | 28 | Susc | Susc |  |
| **AF Turkey Mean** | 27.3 | 21.3 | 27.3 | 18.7 | 23.3 | 24 | 26.7 | 25.3 |  |  |  |
| **AF Turkey SE** | 1.8 | 4.7 | 1.8 | 4.7 | 1.8 | 3.6 | 0.67 | 2.67 |  |  |  |

Disk diffusion results are listed for all antibiotics except for oxacillin and vancomycin, for which standard inhibitory concentrations are listed. Dark Grey = resistant, light grey = intermediate, white = susceptible, black = MDR isolate. Those with complete resistance to at least three antibiotics were considered “multiple drug-resistant” (MDR). AFC=antibiotic-free chicken, AFT=antibiotic-free turkey, Cld=clindamycin, Tet=tetracycline, Cef=cefotaxime, Ery=erythromycin, Chl=chloramphenicol, Gen=gentamicin, Cip=ciprofloxacin, Rif=rifampin, Oxa=oxacillin, Van=vancomycin, SE=standard error, Susc=susceptible, Inter=intermediate resistance, Resis=complete resistance. All values for disk diffusion are in millimeters, which reflect the diameter of the growth ring around the antibiotic disk.
